# Therapeutic plasma exchange decreases serum triglyceride level rapidly and reduces early recurrence rate but no advantages in improving outcomes for patients with hyperlipidemic acute pancreatitis: a retrospective propensity score matching analysis based on twenty year’s experience

**DOI:** 10.1186/s12902-024-01562-z

**Published:** 2024-03-06

**Authors:** Canbin Zheng, Yongping Zheng, Zihui Zheng

**Affiliations:** 1 Department of Endocrine and Metabolic Disease, Shantou Central Hospital, Shantou, Guangdong China; 2 Department of Gastroenterology, Shantou Central Hospital, 114 Waima Road, Shantou,, 515031 Guangdong China; 3 Department of Anesthesiology, Shantou Central Hospital, Shantou, Guangdong China

**Keywords:** Hyperlipidemic acute pancreatitis, Therapeutic plasma exchange, Propensity score matching, Recurrence, Local complications

## Abstract

**Background:**

Hyperlipidaemic acute pancreatitis (HLAP) has become the most common cause of acute pancreatitis (AP) not due to gallstones or alcohol (Mosztbacher et al, Pancreatology 20:608-616, 2020; Yin et al, Pancreas 46:504-509, 2017). Therapeutic plasma exchange (TPE) has been reported to be effective in reducing serum TG levels which is important in management of HLAP (World J Clin Cases 9:5794-803, 2021). However, studies on TPE are mostly focusing on cases reports, TPE remains poorly evaluated till date and need to be compared with conservative therapy with a well-designed study.

**Methods:**

A retrospectively cohort study on HLAP patients between January 2003 and July 2023 was conducted. Factors correlated with efficacy of TPE were included in a propensity model to balance the confounding factors and minimize selection bias. Patients with and without TPE were matched 1:2 based on the propensity score to generate the compared groups. Lipid profiles were detected on admission and consecutive 7 days. The triglyceride (TG) level decline rates, percentage of patients to reach the target TG levels, early recurrence rate, local complications and mortality were compared between groups.

**Results:**

A total of 504 HLAP patients were identified. Since TPE was scarcely performed on patients with TG < 11.3 mmol/L, 152 patients with TG level 5.65 to 11.3 mmol/L were excluded while 352 with TG ≧11.3 mmol/L were enrolled. After excluding 25 cases with incomplete data or pregnancy, 327 patients, of whom 109 treated without TPE while 218 treated with TPE, were included in data analysis. One-to-two propensity-score matching generated 78 pairs, 194 patients with well-balanced baseline characteristics. Of 194 patients enrolled after matching done, 78 were treated without while 116 with TPE. In the matched cohort (*n* = 194), patients treated with TPE had a higher TG decline rate in 48 h than those without TPE (70.00% vs 54.00%, *P* = 0.001); the early recurrence rates were 8.96% vs 1.83%, *p* = 0.055. If only SAP patients were analyzed, the early recurrence rates were 14.81% vs 0.00% (*p* = 0.026) respectively. For patients with CT severity index (CTSI) rechecked within 14 days, early CTSI improment rate were 40.90% vs 31.91%. Local complications checked 6 months after discharge were 44.12% vs 38.30%. Mortality was 1.28% vs 1.72%. No differences were found in early stage CTSI improment rate (*P* = .589), local complications (*P* = .451) or motality between two groups.

**Conclusions:**

TPE reduces TG levels more quickly in 48 h compared with those with conservative treatment, but no difference in the consecutive days. TPE tends to reduce the early recurrence rate comparing with conventional therapy, but TPE has no advantages in improving CTSI in early stage, and no improvement for outcomes including local complications and mortalty.

## Introduction

Acute pancreatitis (AP) is the inflammation of the pancreas with an incidence ranging from 30 to 40 per 100,000 [1–3]. Clinically, there are many causes of AP. Gallstone is the primary cause globally, whereas in China, incidence of hyperlipidemic acute pancreatitis (HLAP) has been reported to be increasing and become the most common cause not due to gallstones or alcohol [4]. A previous study published in 2016 revealed that HLAP accounted for 2–5% of AP cases in the US [3].

HLAP is defined as fasting serum triglyceride (TG) > 11.3 mmol/L or serum TG levels 5.65–11.3 mmol/L along with a visible chylomicrons in the blood [5]. Compared with biliary pancreatitis and alcoholic pancreatitis, HLAP is reported to be more serious and have a higher possibility to develop complications with a poor prognosis [6, 7]. The pathogenesis of HLAP is still unclear, although hydrolysis of TGs and release of free fatty acids have been reported to be the factors correlated [8].

The management of HLAP is mainly supportive with emphasis on lipid-lowering treatment*.* Since first described by Betteridge et al., therapeutic plasma exchange (TPE) has become one of the modilites in treatment of HLAP [9]. TPE is a procedure that removes plasma from the blood and replaces it with new plasma. But TPE is expensive and involved in the use of blood product thus with a potential risk [10]. We have publiched a review analyzing the efficacy and the controversy of TPE [11]. In this study, we retrospectively collect data of HLAP patients admitted in our unit from January 2003 to July 2023, to compare the efficacy of TPE with conventional therapy. The TG decline rates, the early recurrence rate, the CT severity index (CTSI), the incidence of local complications and mortality were analyzed. Propensity score matiching analyzed with R package was performed to reduce the bios due to the confounding factors.

## Patients and methods

### Patients

This is a retrospective cohort study. We continuously analyze 5715 consecutive hospitalized patients who were diagnosed with AP between January 1st, 2003 and July 1st, 2023 at Shantou Central Hospital, Guangdong, China. We review the eletronic charts and those ages ≥ 14 years hospitalized with diagnosis of AP were included. Laboratory parameters, imaging results and outcomes were extracted and those had TG concentrations ≧11.3 mmol/Lwere analyzed. To minimize the selection bios and for balancing the confounding factors, a propensity score matching analysis was performed and patients with balanced basal characteristics were set for further data analysis [12].

### Diagnosis criteria

The diagnostic criteria were based on the 2012 classification of the International Association of Pancreatology [13]. The diagnosis of AP was confirmed by the presence of at least 2 of the following: (1) history and physical exam consistent with the diagnosis; (2) serum lipase or amylase ≥ 3 times the upper limit of normal, or (3) evidence of pancreatitis on image examinations of the abdomen. Diagnosis of HLAP was confirmed according to the aforementioned criteria with a TG level ≥ 11.3 mmol/L (1000 mg/dl) or TG levels 5.65–11.3 mmol/L (500-1000mg/dl) along with visible chylomicrons in the blood after excluding biliary diseases, alcohol, drugs and other factors [13].

Patients with recurrent episodes of AP were included only once. To analyze the dynamic changes, TG levels should have been re-checked at lease once during the hospital stay.

The exclusion criteria include: (1) Biliary, alcoholic, toxic, immune, idiopathic origin; (2) Pancreatic tumors or other malignancy; (3) Chronic diseases: including chronic pancreatitis, chronic organ failure; (4) incomplete clinical data, pregnancy, or loss of follow-up.

### Treatment options

All patients received conventional supportive treatment, including fasting, analgesia, acid inhibition, enzyme inhibition, fluid resustation and antibiotics if needed. Lipid lowering agents were used once patients can tolerate oral intake. In our unit, data showed patients were treated only with fenofibrates in early stage of the disease, no ciprofibrate, gemfibrozil, rosuvastatin or omega-three fatty acids were applied for patients in their acute phases. Initiation of TPE was decided by the attending doctor.

### Procedure of TPE

We performed TPE with Gambro Dialydatoren Hemoselect with 5% albumin as the replacement fluid. After one TPE procedure, the rescure TPE sessions were determined by the attending doctor based on the TG level and clinical manifestations.

### Clinical features

#### Severity assessment

AP severity was classified into 3 grades: severe pancreatitis (SAP), moderately severe pancreatitis (MSAP), and severe pancreatitis (SAP). SAP was defined by the presence of persistent (≥ 48 h) organ failure and/or death. MSAP by the presence of transient (< 48 h) organ failure or local or systematic complications in the absence of persistent organ failure. MAP by the absence of organ failure and/or pancreatic necrosis [13].

### Clinical outcome

#### Therapeutic indicators

TG levels, including day 1, day 2, day 3 and day 5 TG decline rates, time to reach the target TG levels. Outcome indicators: including CTSI changes in early stage, early recurrence events, local complications, therapy-associated complications, the length of stay (LOS) and mortality were monitored.

### Lipid profiles monitor

Lipid profile including serum TG, cholesterol (TC), very low density lipoprotein (VLDL), and low density lipoprotein (LDL) was measured by homogeneous assays carried out by the Beckman Coulter AU5800 autoanalyzer. Serum TG levels < 5.65 mmol/L served as the treatment target level. If available, daily serum TG concentrations were collected until day 14. We found that serum TG levels had not been checked daily in some patients. But 70% of patients in the TPE group and 65% of patients in the n-TPE group had TG levels checked for at least 3 distinct days.

### Follow-up and definition of early recurrence, local complications

Follow-up was carried out through telephone or admission notes until September, 2023, with the emphasis on recurrence events and local complicaitons. We define the early recurrence as the patients developed symptoms and re-hospitalized with diagnosis of HLAP within 3 month’s interval after discharge and only those hospitalized in recent 5 years were collected for early recurrence events analysis.

Local complications of AP were evaluated by two experienced radiologists who were blinded to detailed information about the cases. Acute peri-pancreatic fluid collection (APFC), acute necrotic collection (ANC), pancreatic pseudocyst, and walled-off necrosis (WON) were defined as local complications. Taking account that our data is based on patients hospitalized with the period as long as 20 years, we only include patients who had CT scan repeated within 6 months (4–6 months) after discharge for local complications comparison between groups.

### Definition of alcohol abuse

An alcohol abuser was defined as those who had drunk at least 40 g/d (20 g/d for female) for over 5 years [14].

### Statistical analysis

A Kolmogorov–Smirnov test was used to determine whether the data were normally distributed. If conticuous variables were normally distributed, it was presented as mean ± SD and compared with t test, if not-normally distbuted, variables were presented as median (25, 75% interquartile ranges) and anlayzed with Mann–Whitney test. For categorical variables, Pearson χ2 test was used for across groups test. IBM SPSS statistics version 22 software (IBM, Armok, NY, USA) was used for statistics analysis. *P* < 0.05 was considered statistically significant.

To minimize the selections bios and gain the basal characteristics balanced, a propensity score matching analyzed with R package (version 4.1.1) was performed. Factors correlated with decision to initiate TPE were set as variables in logistic regression model. The variables selected to generate the propensity score included: TG level, age, sex, BMI and Atlanta severity (mild as 1, moderately severe as 2, severe as 3). To analyze the impact of concomitant treatment on the outcomes, use of fenofibrate was set as one of the covariates when performing PSM.

Since we had more cases treated with TPE than treated with conservative treatment, a 1:2 matching between groups was performed based on the propensity score. With a caliper 0.02, a nearest neighbor and matched without replacement option, we generate two matched groups. Currently, there is no universally agreed standardized difference in indicating a neglibile difference to estimate the relevance of balance between the matched groups; some recommended < 10%, some agured that if the standardized difference between both groups < 20%, their comparability is considered to be good [15, 16].

In the current study, Stddifff.R (calculated with “R” package stddiff version 3.0) was used to calculate the standardized difference after matching. 31 cases in n-TPE and 116 in TPE failed to match. Finally, 116 from TEP group, 78 from n-TPE group were generated and applied for further analyses. When use of fenofibrate was set as covariate in PSM to compare outcomes between groups, only with the caliper set to 0.004 can 72 cases be successfully generated in pairs.

### Sample size estimation

The main purpose of our study is to investigate the TG decline rates. It has been reported that the 24 h deline rate is about 71% [17] if treated with TPE. On the basis of our observation, for those treated with conventional therapy, the 24 h decline rate is about 65%. Assuming the power of 80%, SD = 20%, a = 0.05, the minimum sample size comes out to be 102 (calculated with G-power). We included 230 cases in our study.

## Results

### Patient selection and matching

**Fig. 1:**
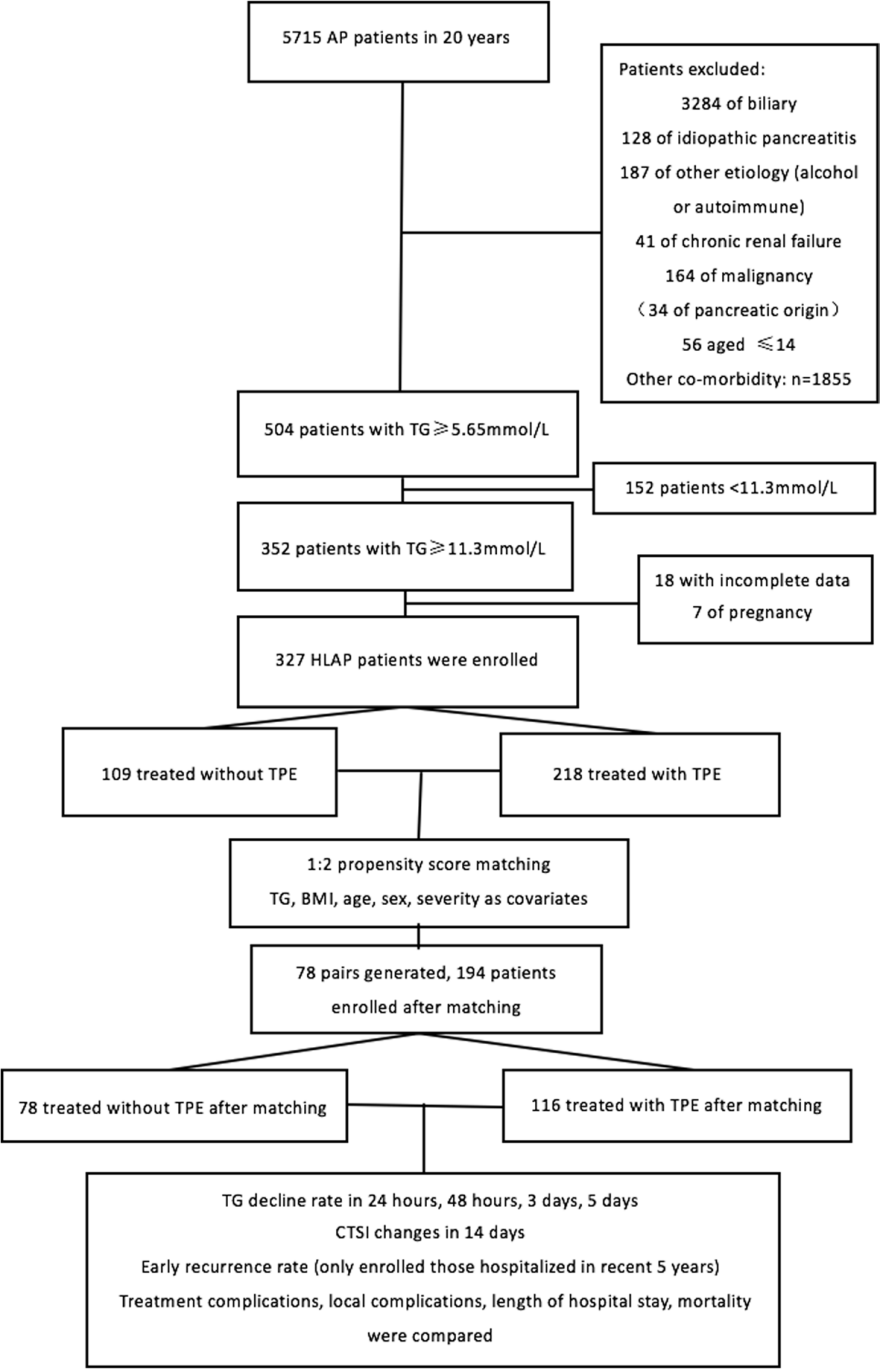
Flow chart of the patient selection procedure

During the 20-years study period, 5715 patients were hospitalized with AP at Shantou Central Hospital. Among these 5715 patients, 504 patients were diagnosed as HLAP defined as TG level ≧ 5.65 mmol/L. After excluding episodes that did not meet the criteria listed above and excluded those TG level < 11.3 mmol/L, 352 patients diagnosed with HLAP with TG level ≧ 11.3 mmol/L were included in the study. Based on the therapy received, we identify 218 patients of TPE group and 109 patients of non-TPE group (flow chart). Figure 1 illustrates the flowchart of the patient selection procedure.

**Fig. 2:**
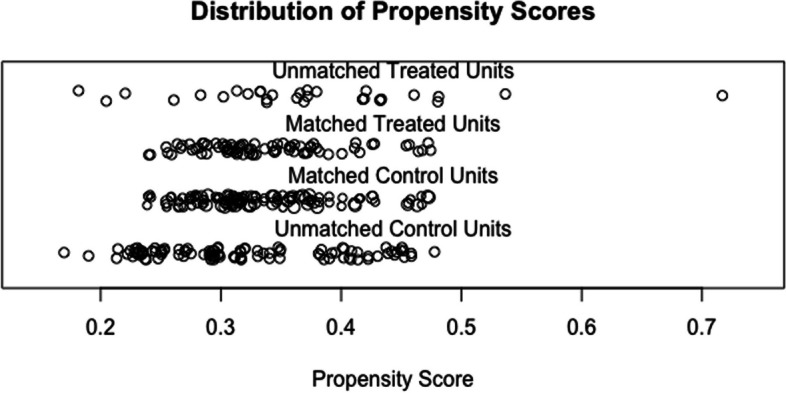
Distribution of propensity scores

After propensity score matching, there were 117 patients in TPE group and 78 in non-TPE group. All five confounding variables had an acceptable standardized difference (< 20%), indicating that the matching procedure was efficient in creating a balance between the TPE and n-TPE groups (Figs. 1 and 2).

Patients in the n-TPE and TPE groups had mean propensity scores of 0.3297 (95% confidence interval (CI) = 0.3198 to 0.3395) and 0.3396 (95% CI = 0.3246 to 0.3545), respectively, Using the algorithm described above, we were able to match 178 patients in the TPE group with 98 in the n-TPE group (Fig. 2).

**Fig. 3:**
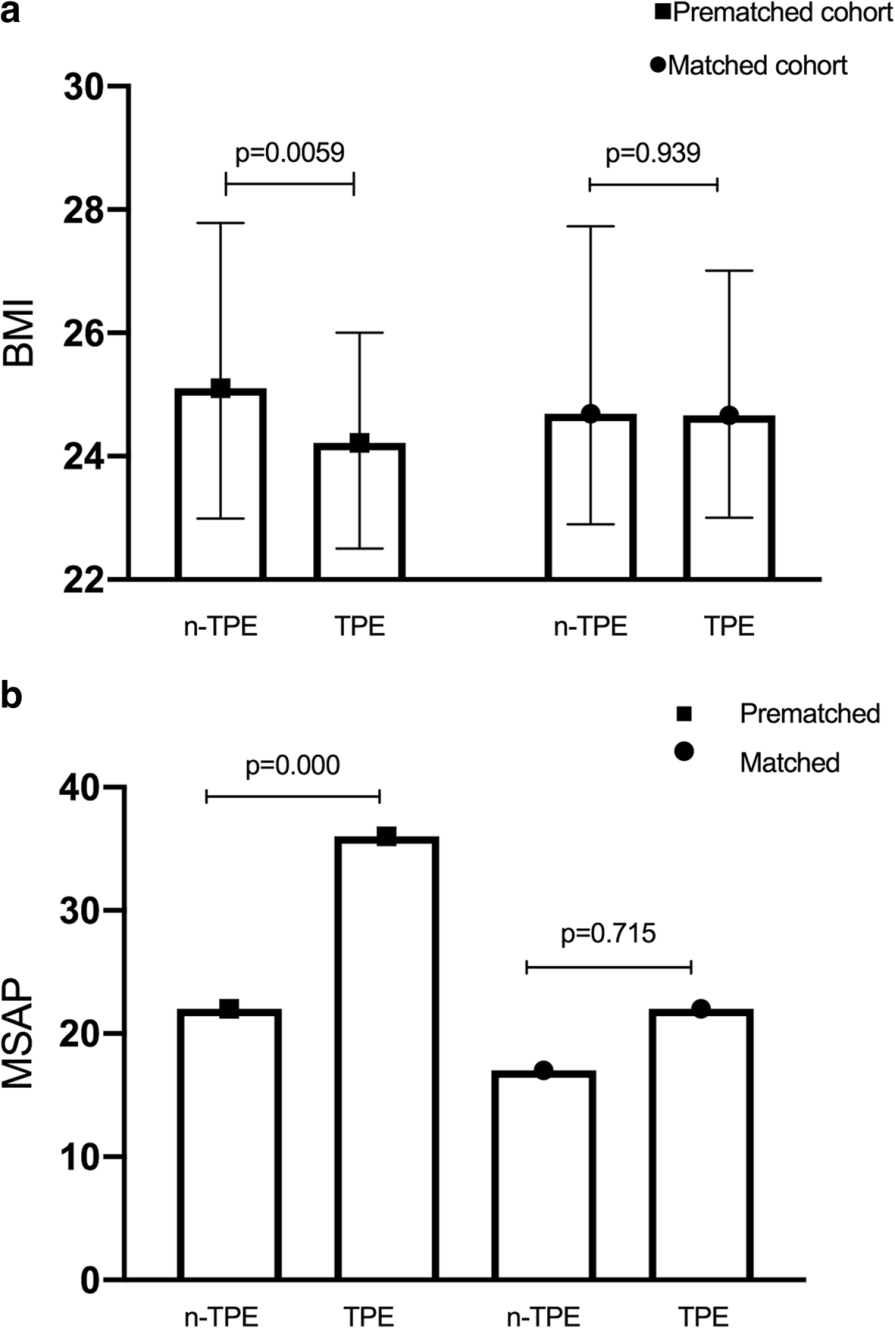
BMI and types of AP before and after matching. a demonstrates that patients in the TPE group had a higher TG level (*p* = 0.002). **a** possible higher BMI (*p* = 0.059) and gain balance after matched. **b** showed a higher proportion of MSAP (*p* = 0.000), and gain balance after matched

The characteristics of all patients in the n-TPE and TPE groups are shown in Table 1. Analysis of the total patients showed that patients in the TPE group had a higher TG level (*p* = 0.002), a possible higher BMI (*p* = 0.059), a higher proportion of MSAP (*p* = 0.000), and gain balance after matched (Table 1, Fig. 3a, b).

**Table 1:** Patient characteristics in total and matched cohorts

**Characteristic**	**Total set**			**Matched set**		
	**n-TPE** **(*n* = 109)**	**TPE** **(*n* = 218)**	***p***	**n-TPE** **(*n* = 78)**	**TPE** **(*n* = 116)**	***p***
**Age, yr**	38 {32.5, 45}	37 {31, 45}	0.462	38 {32, 45}	37 {31, 45}	0.715
**Sex, male%**	74 (67.89%)	164 (75.23%)	0.188	53 (67.95%)	86 (74.14%)	0.417
**BMI**	25.10 {22.99, 27.78}	24.22 {22.57, 26.60}	0.059	24.69 {22.90, 27.73}	24.66 {23.00, 27.01}	0.939
**TG**	16.76 {13.75, 20.07}	18.17 {16.08, 21.55}	0.002^a^	17.29 {13.64, 21.24}	17.81 {15.77, 20.30}	0.184
**Atlanta**						
SAP, n%	36 (33.28%)	91 (41.73%)	0.149	31 (39.74%)	44 (37.93)	0.881
MSAP, n%	22 (20.18%)	36 (16.51%)	0.000	17 (21.79%)	22 (18.97%)	0.715
MAP, n%	51 (46.79%)	91 (41.74%)	0.409	30 (38.46%)	50 (43.10%)	0.554
**Coexisting disease (n %)**						
DM	80 (73.39%)	177 (81.19%)	0.117	59 (75.64%)	94 (81.03%)	0.376
Alcoholic	23 (21.10%)	56 (25.69%)	0.412	19 (24.36%)	30 (25.86%)	0.867
**Concomitant treatment**						
Fenofibrate (started within 5 days of onset)	55.46% (60/109)	49.08% (107/218)	0.348	57.69% (45/78)	49.14% (57/116)	0.240
Fenofibrate (started after 5 days of onset)	8.26% (29/109)	27.06% (59/218)	1.000	32.05% (25/78)	25.86% (30/116)	0.417
**Standardized difference**^**b**^						
	Pre-matched (95% CI)			Matched (95% CI)		
TG	0.009 (-0.221–0.239)			0.106 (-0.181–0.394)		
BMI	0.183 (-0.047–0.414)			0.012 (-0.275–0.299)		
Age	0.075 (-0.155–0.305)			0.073 (-0.214–0.360)		
Sex	0.163 (-0.068–0.393)			0.137 (-0.151–0.424)		
Severity	0.184 (-0.047–0.414)			0.1 (-0.187–0.388)		

Values are expressed as Median {25th, 75th percentile} or number (%)

*DM* diabetes mellitus

^a^*p* < 0.01 between two groups

^b^calculated with “R” package stddiff version 3.0

### TG level changes in different groups

The TG concentrations declined rapidly in both groups. The fall of TG levels in the TPE group and n-TPE group is showed in Table 2.

**Table 2:** Percent decrease of TG levels and percentage of patients to reach the target TG levels on each day in n-TPE and TPE groups

	**Day 1**	**Day 2**	**Day 3**	**Day 5**
**Percent decrease of TG levels on each days**				
**Before matched**				
n-TPE group	57.00 {24.00, 72.00} *n* = 37	54.00 {42.75, 72.00} *n* = 34	67.00 {55.00, 78.00} *n* = 31	77.50 {63.25, 84.75} *n* = 56
TPE group	55.00 {31.50, 74.50} *n* = 117	70.00 {57.75, 79.00} *n* = 94	75.50 {61.00, 80.75} *n* = 100	80.00 {68.25, 86.00} *n* = 96
*P* value by Mann–Whitney	0.906	0.001	0.143	0.185
**After matched**				
n-TPE group	60.00 {34.00, 74.50} *n* = 29	58.50 {41.50, 75.00} *n* = 22	68.50 {55.25, 82.00} *n* = 24	78.00 {68.00, 85.25} *n* = 46
TPE group	56.00 {28.50, 68.75} *n* = 60	69.00 {55.75, 79.00} *n* = 54	68.50 {55.50, 82.00} *n* = 54	79.00 {65.50, 86.00} *n* = 43
*P* value by Mann–Whitney	0.367	0.077	0.918	0.736
**Percentage of patients to reach the target TG levels**				
**Before matched**				
n-TPE group	21.62% (*n* = 37)	23.53% (*n* = 34)	45.16% *n* = 31)	62.50% (*n* = 56)
TPE group	26.09% (*n* = 115)	57.89% (*n* = 95)	63.27% (*n* = 98)	71.88% (*n* = 96)
*P* value by Pearson χ2 test	0.667	0.001	0.094	0.230
**After matched**				
n-TPE group	27.59% (*n* = 29)	27.27% (*n* = 22)	50.00% (*n* = 24)	63.04% (*n* = 46)
TPE group	28.33% (*n* = 60)	52.73% (*n* = 55)	59.26% (*n* = 54)	62.79% (*n* = 43)
*P* value by Pearson χ2 test	0.941	0.043	0.447	0.980

*TPE* therapeutic plasma exchange

Percent changes were calculated only in patients who had TG concentration available for that day

The number of subjects with paired data for each day is also shown

Median {25th, 75th percentile} TG concentrations in n-TPE and TPE groups

**Fig. 4:**
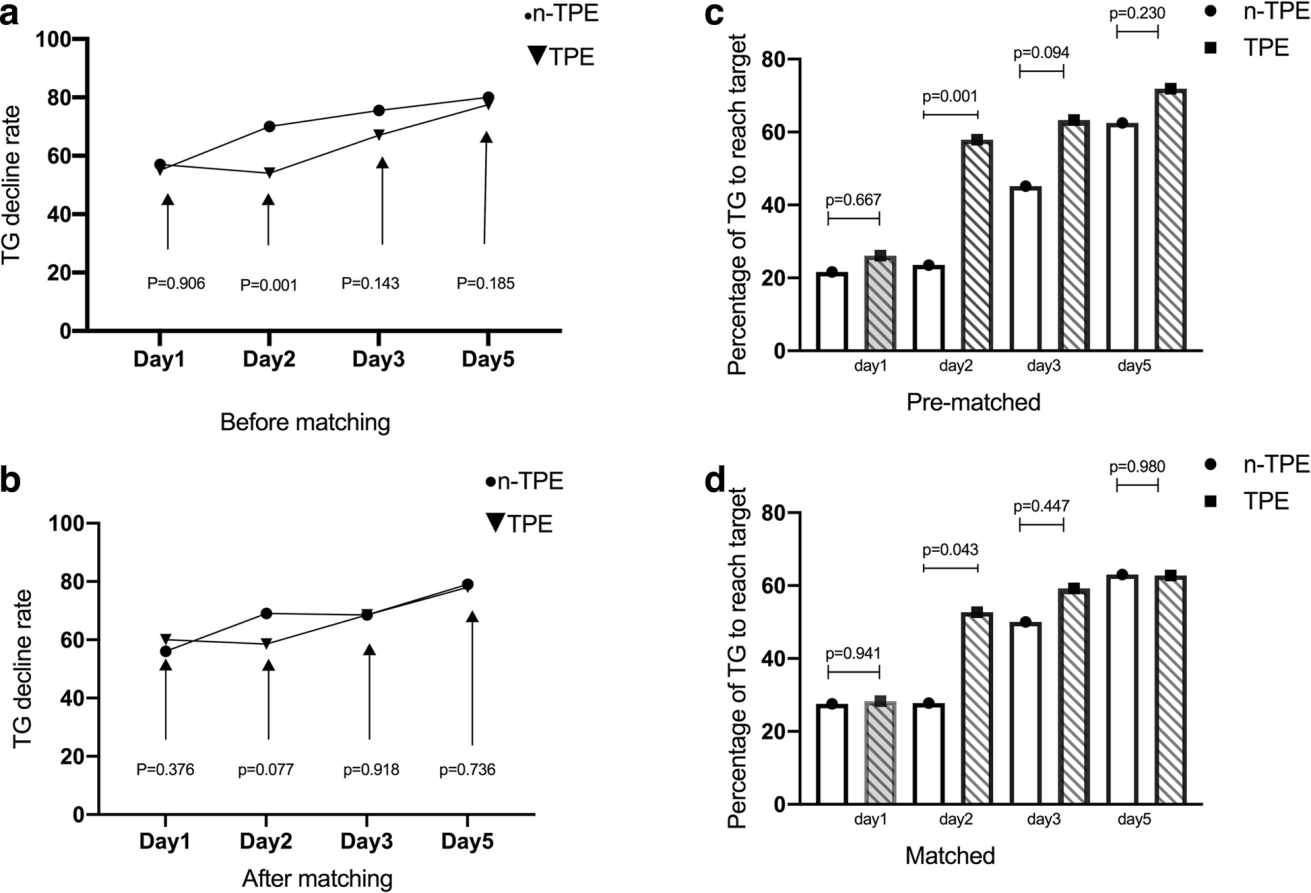
Dynamic changes of TG levels and percentage of patients to reach the target TG levels in n-TPE and TPE groups (**a**-**d**)

Figure 4a demonstrates that TG declined in both groups. The difference between groups had significant difference in day 2 before matching. Figure 4b shows TG level declined in both group, with a higher TG decline trend in group TPE (*p* = 0.077) after matching. Figure 4c shows the percentage of patients to reach the target TG level (TG < 5.65 mmol/L) was higher in group TPE in day 2 before matching (*p* = 0.001). Figure 4d shows a higher percentage of patients in group TPE to reach the target TG level in day 2 after matching (*p* = 0.043).

### Outcomes in different groups

#### CTSI changes within 14 days

Only patients with their CT rechecked with an interval of 3–14 days after initiation of treatment were included. If the patient underwent more than two CT scan, the earliest CT result was analyzed. The results show there is no significant difference between groups Table 3.

**Table 3:** Outcomes in pre-matched and propensity-matched groups

	**Pre-matched cohort**			**Matched cohort**		
	**n-TPE** **(*n* = 109)**	**TPE** **(*n* = 218)**	***p***	**n-TPE** **(*n* = 78)**	**TPE** **(*n* = 116)**	***p***
**CTSI changes in 14 days**						
	*n* = 25	*n* = 57		*n* = 22	*n* = 47	
Improved	13 (52%)	20 (35.09%)	0.221	9 (40.90%)	15 (31.91%)	0.589
Deteriorated	11 (44%)	30 (52.63%)	0.632	9 (40.90%)	26 (55.32%)	0.309
Unchanged	1 (0.04%)	7 (12.28%)	0.424	4 (18.18%)	6 (12.77%)	0.715
**Early recurrence (in 3 months)**						
All type cases	8 *n* = 84 (9.52%)	4 *n* = 161 (2.48%)	0.025	6 *n* = 67 (8.96%)	2 *n* = 109 (1.83%)	0.055
Only SAP included	6 *n* = 36 (16.66%)	2 *n* = 91 (2.19%)	0.007	4 *n* = 27 (14.81%)	0 *n* = 44 (0.00%)	0.026
**Treatment complications**						
Venous embolism				0	6	0.083
**Local complications**						
	15 (13.76%)	43 (19.72%)	0.117	12 (15.38%)	24 (20.69%)	0.452
**Median length of stay, days (IQR)**						
All type cases	8 (6, 10)	11 (6, 15)	0.000	8 (6, 11)	11 (8, 15)	0.000
SAP	8.5 (6.25, 11)	13 (9, 18)	0.000	9 (7, 11)	13 (9.25, 18)	0.001
**Mortality**						
				1 (1.28%)	2 (1.72%)	1

*SAP* severe acute pancreatitis, *MSAP* moderately severe acute pancreatitis, *MAP* mild acute pancreatitis, *DM* diabetes mellitus, *IQR* Interquartile range

#### Early recurrence

We define the early recurrence and analyze those only recurred within 3 months after discharge. The result reveals the early recurrence rate is higher in group of n-TPE, particularly when SAP patients were analyzed after matching (14.81% vs 0.00%) (Table 3).

#### Incidence of local complications

Because of the long time span of the study, we only collect and analyze those had their image re-checked within 6 months after discharge. The result demonstrates that incidence of local complications has no difference between groups (15.38% vs 20.69%, *p* = 0.452 after mathing) Table 3.

#### Treament complications

No vascular complications were noted in n-TPE, while there were 6 cases that developed venous complications in group of TPE Table 3.

#### Length of hospital stay

Length of hospital stay was significantly longer (*P* < 0.001) in matched PE group than in the matched n-TPE group Table 3.

#### Mortality

Among all 276 eligible patients after propensity-matching, the mortality was 3.06% (3/98) vs 1.12% (2/178), there was no significant difference between two groups, *P* = 0.248 Table 3.

### Outcomes in different groups when use of lipid lowering agent was included as covariate

Considering the use of lipid lowering agent as the concomitant treatment may have impacts on outcomes, we conduct a propensity score matching with more covariates. TG level, age, sex, BMI, Atlanta severity and use of fenofibrate within 5 days of the onset were set as covariates. Only with the caliper set to 0.004 can we generate 72 cases in pairs with 33 in n-TPE and 39 in TPE groups. We find a higher percentage of TG to target level in group of TPE in 2 days (0.09% vs 68.75%, *p* = 0.046). When comparing those treated concomitantly with or without fenofibrate, patients in group of TPE had a higher percentage to target TG level, indicating that the higher TG decline rate mainly resulted from the effect of TPE rather than from use of fenofibrates. Besides, we find no difference of CTSI improvement, local complications in the matched cohort between groups, inferring there is no advantages of TPE in improving outcomes even when the use of fenofibrate is included as covariate in PSM, Table 4.

**Table 4:** Percentage of TG to target level and outcomes comparison in matched cohort with fenofibrate set as covariate

	**Matched cohort**				
	**n-TPE (*n* = 33)**		**TPE (*n* = 39)**		***p***
**Percentage of TG to target level in 2 days**^a^	0.09% (1/11)		68.75% (11/16)		0.046
	without fenofibrate	with fenofibrate	TPE without fenofibrate	TPE with fenofibrate	
	0.09 (1/11)	0 (0/5)	68.75% (11/16)	16.66% (2/12)	
	*P* = 0.500^b^		*P* = 0.126^b^		
**Percentage of TG to targer level in 3 days**^a^	44.44% (4/9)		68.75% (11/16)		0.912
	without fenofibrate	with fenofibrate	TPE without fenofibrate	TPE with fenofibrate	
	44.44% (4/9)	17.64% (3/17)	50.00% (8/16)	13.33% (2/15)	
	*P* = 0.151^b^		*P* = 0.032^b^		
**CTSI changes in 14 days**					
Improved	61.54% (8/13)		42.11% (8/19)		0.288
Deteriorated	38.46% (5/13)		36.84% (7/19)		0.927
Unchanged	0		21.05% (4/19)		
**Early recurrence (in 3 months)**	1		0		
**Local complications**^c^	31.25% (5/16)		31.58% (6/19)		0.984
**Mortality**	0		0		

^a^TG level < 5.65 mmol/L was set as the target level

^b^compare the group with or without fenofibrate

^c^Only those have their CT rechecked within 6 months included

## Discussion

This study investigated the changes of lipid profiles and clinical outcomes after TPE or conventional treatment in a cohort of HLAP patients. Our data clearly demonstrates that TG levels decreased both in n-TPE and TPE groups, specifically; TG declined more quickly in the second day in TPE group than that in group n-TPE, while there were no differences of the trends regarding the TG decline rate in the consecutive days. Also, we demonstrate that TPE tended to reduce early recurrence rate, but we find no advantages of TPE in improving early CTSI changes, local complications and mortality compared with conventional treatment.

AP is the inflammation of pancrease with > 270,000 cases reported annually in the US [18]. Causes of AP include gallstone, alcohol, and hyperlipidemia. According to a a report published in 2017, hyperlipidemia has exceeded alcohol to become the second major cause of pancreatitisin China [4]. This trend is believed to be associated with the improvement of the living standard and the change of diet structure [19]. As in our data, for patients hospitaliszed in 20 years, biliary diseases remained the first, while HLAP has become the second major cause. Our data revealed that HLAP accounted for 8.81%, consistent with reports by other authors [4, 19].

Clinically, there are many available therapies for HLAP. Oral lipid-lowering agents, intravenous insulin, and TPE have been published to have the efficacy in lowring TG levels [20–23]. The possible mechanism of HLAP is that the lipoprotein substrates and excess free fatty acid molecules damage the microvascular membrane of pancreatic latent acinar cells [24]. Thus, quickly decreasing TG level is imperative. Oral lipid lowering agents should be started when a patient is able to tolerate oral intake. TPE was first described by Betteridge et al. [18]. It has been reported that TPE rapidly removes TG from the blood and effectively reduces the levels of pancreatic enzymes, eliminates pro-inflammatory cytokines, including interleukin-1 and tumor necrosis factor-α [5, 25].

But TPE has a relatively high treatment cost and potential risks [10]. Reports have demonstrated that infusion of insulin and heparin [26] in reducing TG levels. Besides, some authors argue that fasting often leads to a significant reduction in TG concentrations [27]. Thus, the efficacy of the treatment is still controversial. In 2016, the American Society for apheresis suggested that HLAP should only be regarded as a Class III indication for plasma exchange (PE) [28], implying that the efficacy of TPE is not established and candidates screening should be individualized. We have publiched a review analyzing the efficacy and the controversy of TPE [11]. In the current study, we focus on the TG decline rates as well as the impact on the outcomes.

Since TPE has become a standard therapy for HLAP in our unit (as well as many unites in China) around the clock. Owing to the ethics reason, RCT is difficult to perform. In order to minimize the seletion bios, we perform the propensity score matching. Clinically, doctors tend to apply TPE for patients with a higher TG level and a suspected more severe conditions. In our data, TG levels, proportion of MSAP in TPE group was significantly higher than group of n-TPE. Only when the caliper be set to 0.02 can you get TG and severity classification balanced after mathing, indicating the obvious inbalance of TG level in pre-matched cohort. Up to 133 cases fail to match but be abandoned in no choice. In order to elucidate the impact of concomitant treatment on outcomes, we compare the use of lipid lowering agent in different groups. We find no significant difference of the proportion of the use of fenofibrate either started within 5 days or after 5 days of the onset and the higher TG decline rates mainly resulted from the effect of TPE rather than from use of fenofibrates. Besides, we find when use of fenofibrate was set as covariate in PSM, no significant difference is observed in CTSI improvement or local complications, implying that the impact of lipid lowering agent can be ignored.

The current study demonstrates a more rapid decline of TG level in the second day after TPE was initiated when compared with n-TPE, but our data find no difference in the consecutive days. This is consistent with other reports. In most patients, TG levels decrease by a percent of 65% to 85% after one or two TPE sessions respectively [5]. Unfortunatly, since our study comprises patients within 20 years, some data might not be convincely enough analyzed whether the time TPE iniated would have influences on the results.

HLAP has more complications, longer hospital course and a higher possibility to recur. HLAP has been reported to have a recurrence rates of approximately 16.0%–49.2% [29, 30]. Recurrent AP (RAP) infers two or more AP attacks in a period of at least 3 months, during which the patients have recoved or nearly recover [31]. The factors affecting recurrence of HLAP is not included in our study design, but we accidently find that the early recurrence rate (recure within an interval of 3 months) is higher in n-TPE groups. Previous studies have shown that smoking, high levels of TG, and substandard control of TGs are risk factors for recurrence of HLAP [14, 29]. Since early recurrence poses a small number of cases, we do not focus on the reason why, but, the results warrant further investigation.

When comparing the early CTSI changes, the local complications, we find no difference between groups. Many factors may have impacts on the severity and outcome of AP [32]. Till date, which patients can benefit from TPE have not been conclusively shown. In the current study, the in-hospital mortality rates were similar in the n-TPE group and TPE groups (1.28% vs. 1.72%; 95% confidence interval, 0.000–0.000, *P* = 0.000). Consistent with the report by Yu and Kichloo [5, 33]. Not to our surprise, TPE group was associated with significantly longer median hospital stay (8 vs 11 days, *P* < 0.000).

Previous studies have revealed that TPE is associated with complications, including allergy, infection, unstable circulation and abnormal blood coagulation [34]. We have published a paper revealing a deep venous thrombus post TPE [35]. In the current study, we find 6 patients who developed venous thrombus. Taking accout the high cost, the uncertain efficacy, the possible complications, a careful candidate selection for TPE treatment is needed.

In summary, TPE dicreases TG level more quickly than conventional therapy in early days, but exerts no effects on improving early CTSI changes, local complications, or mortality. The possibility of TPE in reducing the early recurrence rate demonstrated in the current study warrants further investigation.

### Limitations

This is the first study which includes data based on 20 year’s experience with more than 300 cases. However, there were several limitations of this study. First, this was a retrospective analysis, not a prospective design. The propensity socre matching can not overcome all the bias. Besides, only with the caliper 0.02 can we organize the paired cases, this can make half of the cases be abandoned and out of the analysis. Second, the included patients come from a period as long as 20 years, although we only collect patients in recent 5 years for early reccurence rata analysis, only those had their CT scan within 6 months after discharge were included for local complications comparison, missing data cannot be totally avoided. Third, we accidently find the early reccurence rate was lower in group TPE, because of the small sample size, this can only be confirmed in later study.

## Conclusions

On the basis of our propensity score analysis on data from 20 year’s experience, we find that TPE exerted a more rapid decline of TG levels in early days than conventional therapy. Besides, we find TPE tended to reduce the early reccurence rate, but, we find TPE exerted no advantages in improving CTSI, local complications or mortality for HLAP patients.

## Data Availability

The data generated during and/or analysed during the current study are available from the corresponding author on reasonable requese.
